# Development of Spinal Tuberculosis in an Adolescent With Crohn's Disease After Infliximab Therapy: A Case Report With Literature Review

**DOI:** 10.3389/fped.2021.802298

**Published:** 2022-02-09

**Authors:** Jae Hoon Jung, Sujin Choi, Youra Kang, Dae-Chul Cho, So Mi Lee, Tae In Park, Byung-Ho Choe, Dongsub Kim, Ben Kang

**Affiliations:** ^1^Department of Pediatrics, School of Medicine, Kyungpook National University, Daegu, South Korea; ^2^Department of Neurosurgery, School of Medicine, Kyungpook National University, Daegu, South Korea; ^3^Department of Radiology, School of Medicine, Kyungpook National University, Daegu, South Korea; ^4^Department of Pathology, School of Medicine, Kyungpook National University Daegu, South Korea

**Keywords:** Pott's disease, Crohn's disease, tuberculosis, tumor necrosis factor, infliximab, vedolizumab

## Abstract

**Introduction:**

Tuberculosis (TB) spondylitis, also known as Pott's disease, is a severe form of extrapulmonary TB. Infliximab treatment for Crohn's disease (CD) patients increases the risk of TB, and is likely to increase the risk of TB spondylitis as well.

**Case Presentation:**

We report a rare case of TB spondylitis development in a 16-year-old female with CD. She had a close household contact of active pulmonary TB and received contact investigation. She was diagnosed with latent TB 1 month before the diagnosis of CD, and had started a latent TB treatment regimen with isoniazid for 9 months. At 5 months from the start of latent TB treatment, infliximab was started. Approximately 1 year after infliximab treatment, her infusion interval was shortened from every 8 weeks to every 4 weeks owing to secondary loss of response due to nonimmunogenic pharmacokinetic failure. One month later, miliary TB developed and infliximab was stopped. She received a miliary TB treatment regimen for 6 months, curing the disease. Three months later, spinal TB was incidentally detected on abdominal computed tomography. She received a TB treatment regimen for 12 months, curing spinal TB. Currently, she is receiving vedolizumab to treat CD and is in clinical remission. Although this patient has sufficiently been treated at each stage of TB development, particularly for latent TB and miliary TB, TB spondylitis still developed.

**Conclusion:**

Considering that TB spondylitis developed despite sufficient treatment at each stage, pediatric gastroenterologists should stay cautious when using anti-tumor necrosis factor agents in patients with inflammatory bowel disease with a history of latent TB.

## Introduction

Crohn's disease (CD) is a chronic relapsing inflammatory bowel disease (IBD) characterized by ulceration and inflammation throughout the gastrointestinal (GI) tract (1). Approximately 25% of patients with CD are diagnosed at <20 years of age, in whom the disease is more severe and aggressive compared with adult-onset disease. Therefore, children and adolescents with CD may require an earlier introduction of immunomodulators and/or biologics (1–3).

Tuberculosis (TB) spondylitis, also known as Pott's disease, is a severe form of extrapulmonary TB. In about 1% of all TB infections, venous spread of *M. tuberculosis* results in vertebral infection (4, 5). Possible complications are spinal deformities, such as growth plate destruction, kyphosis, and spinal column destruction, followed by neurological complications. Anti-tumor necrosis factor (TNF) treatment inhibits the phagocytic activity of macrophages, which delays granuloma formation (6). Thus, anti-TNF treatment for patients with CD increases the risk of reactivation and disease progression of TB, but its impact on TB spondylitis is uncertain (7). For active pulmonary or extrapulmonary TB patients, at least 2 months of TB treatment is required before starting anti-TNF treatment, and for latent TB patients, at least 4 weeks of TB treatment is required (7, 8).

Herein, we report a rare case of an adolescent with CD in whom spinal TB developed during the treatment with infliximab (IFX).

We present the following article in accordance with the CARE reporting checklist.

## Case Presentation

A 16-year-old female was admitted to Kyungpook National University Children's Hospital with complaints of diarrhea, hematochezia, and weight loss for a year. She had been diagnosed with latent TB 1 month before her visit, and a latent TB treatment regimen with isoniazid (INH) for 9 months had been started. She had completed Bacillus Calmette–Guérin (BCG) vaccination. Family history revealed that her father had a history of pulmonary TB, which was treated 3 years ago.

**Figure 1:**
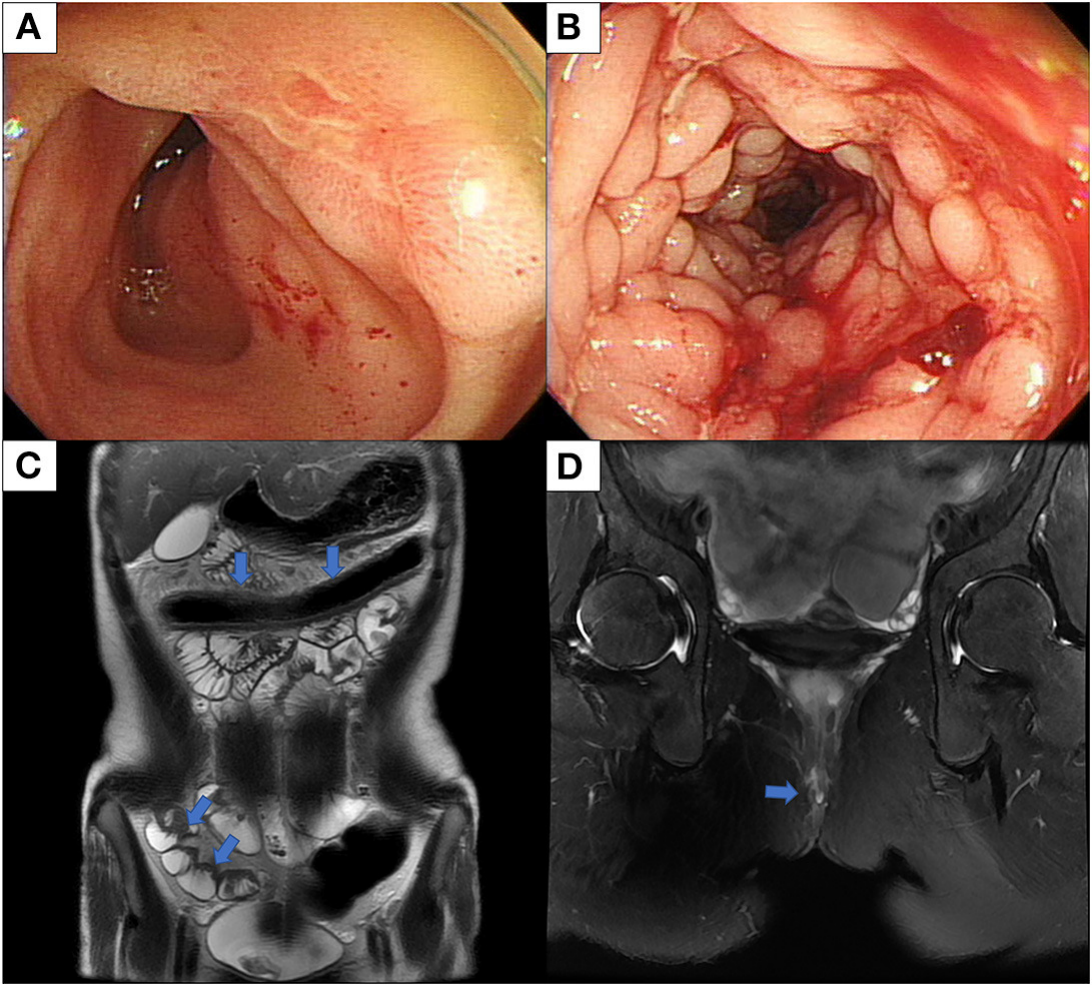
Ileocolonoscopy and MRI conducted at diagnosis of Crohn's disease. **(A)** Ileocolonoscopy revealed an ulcer in the terminal ileum, and **(B)** linear deep ulcers with cobblestoning in the transverse colon. **(C)** MRE shows multifocal asymmetric bowel wall thickening involving the transverse colon, terminal ileum, and jejunum and luminal narrowing in the transverse colon with diffusion restriction and enhancement. **(D)** MRI of the pelvis shows two intersphincteric perianal fistulas. MRI, magnetic resonance imaging; MRE, magnetic resonance enterography.

On admission, vital signs were within normal range. On physical examination, a perianal fistula with the discharge was observed in the perianal area. Initial laboratory tests showed a white blood cell count of 5,130/μL, a hemoglobin level of 11.6 g/dl, a platelet count of 462,000/μL, an albumin level of 3.5 g/dl, an erythrocyte sedimentation rate (ESR) of 99 mm/h, and a C-reactive protein (CRP) of 3.51 mg/dl. The fecal immunochemical test was positive and fecal calprotectin was 589.9 mg/kg. No pathogens were detected in stool culture and stool polymerase chain reaction (PCR). Chest x-ray showed no abnormal findings in the lungs. However, the interferon-gamma release assay was positive. Ileocolonoscopy showed multifocal ulcers throughout the terminal ileum and colon (Figures 1A,B). Histology revealed cryptitis, crypt abscesses, and noncaseating granulomas; however, the acid-fast bacillus smear, culture, and PCR for TB were negative. Magnetic resonance enterography showed multifocal wall thickening in the colon, ileum, and jejunum and two intersphincteric perianal fistulas (Figures 1C,D). Upper GI endoscopy was unremarkable. The patient was diagnosed with CD with a phenotype of A1b, L3+L4b, B1p, G0 according to the Paris classification. Her pediatric CD activity index (PCDAI) score was 50 and the Simple Endoscopic Score for CD (SES-CD) was 26.

Treatment was begun with exclusive enteral nutrition (EEN), mesalazine, and azathioprine, which were effective. Two weeks after the diagnosis, she underwent seton placement. However, 1 month after finishing her 8-week treatment with EEN, her disease relapsed. Azathioprine was changed to methotrexate (MTX). However, symptoms did not improve, and infliximab (IFX) was started 1 month later, considering that she had taken sufficient latent TB treatment with INH for 5 months. The patient chose to take IFX as her biologic agent between IFX and adalimumab, which are the only biologic agents currently approved for the treatment of moderate-to-severe CD. Chest x-ray before starting IFX showed no abnormal findings in the lungs. After starting IFX, INH therapy for latent TB treatment was extended for a total of 12 months.

Ileocolonoscopy at 1-year follow-up after IFX treatment revealed endoscopic healing. PCR for TB was negative again from specimens of the ileum and cecum. However, ESR and CRP gradually increased 1 month after the ileocolonoscopy, and she developed diarrhea. IFX trough level was 2.4 μg/mL, and IFX antibody was negative. Considering the suboptimal trough level and negative anti-drug antibody status of the patient, secondary loss of response due to nonimmunogenic pharmacokinetic failure was suspected. Therefore, the IFX interval was shortened from every 8 weeks to every 4 weeks.

**Figure 2:**
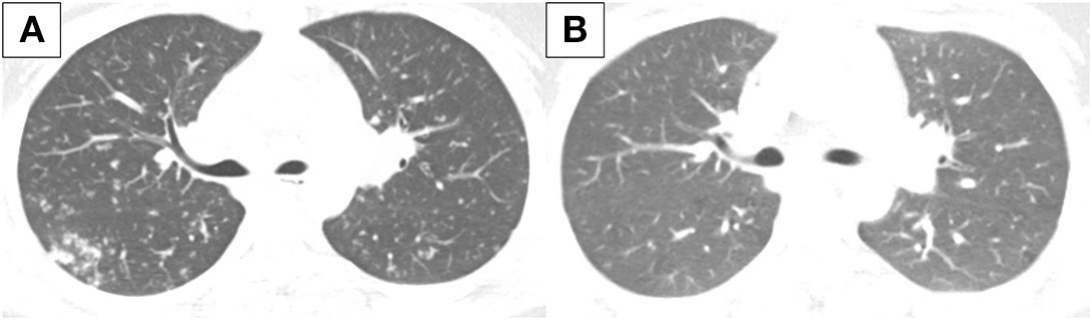
Chest CT conducted **(A)** at diagnosis of miliary TB and **(B)** after TB treatment. **(A)** Innumerable small discrete nodules of random distribution with tree in bud pattern are observed in both lungs. **(B)** There is a decrease in extent of the innumerable small nodules in both lungs. CT, computed tomography; TB, tuberculosis.

However, 1 month later, she newly developed symptoms of fever, cough, and sputum. Chest x-ray and chest computed tomography (CT) showed multiple small nodules in both lungs indicating miliary TB (Figure 2A). Bronchoscopy was conducted, and TB was confirmed by a positive TB PCR test of the tracheal biopsy specimen and bronchial washing fluid. IFX treatment was discontinued and the standard TB treatment regimen with INH, ethambutol, rifampin, and pyrazinamide for 2 months followed by INH, ethambutol, and rifampin for 4 months was started. MTX and mesalazine were maintained to treat CD. Miliary TB was completely cured after 6 months of TB treatment (Figure 2B).

**Figure 3:**
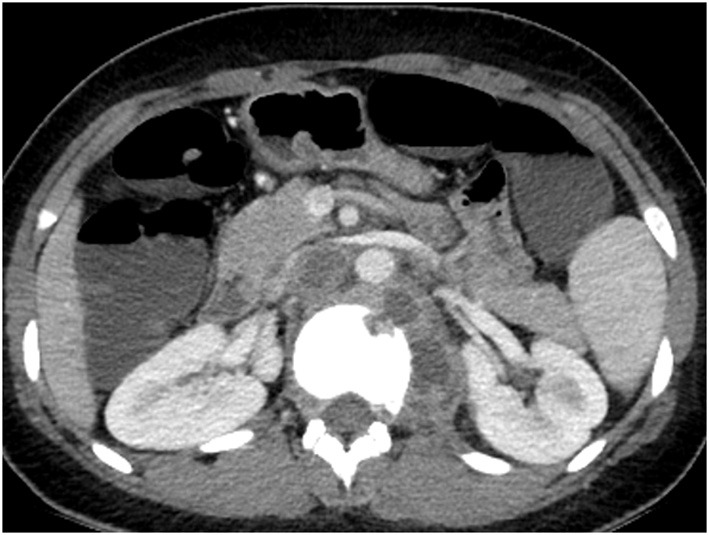
Abdomen CT conducted at 3 months after cessation of miliary TB treatment. A newly defined presumed cold abscess within the left psoas muscle at the level of T10 to L2 and TB spondylitis at the T12 and L1 are observed. CT, computed tomography; TB, tuberculosis.

**Figure 4:**
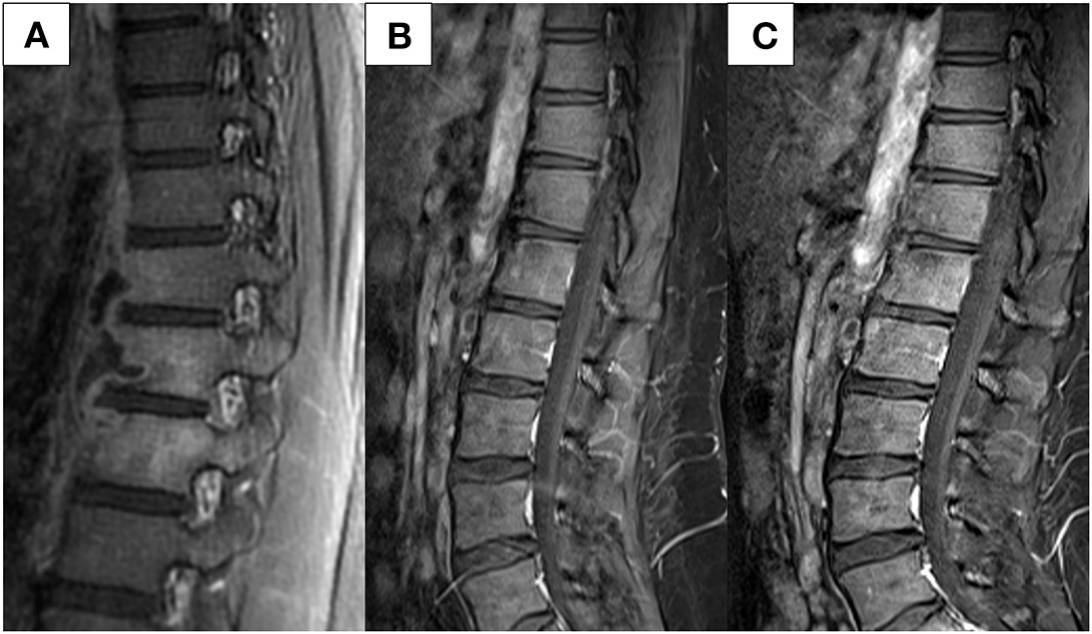
Spinal MRI conducted **(A)** at diagnosis of spinal TB and **(B,C)** follow-up images. **(A)** Bone marrow enhancement at T11-12-L1 with paraspinal fluid collection at left anterolateral side, and relatively thin wall enhancement. **(B)** Improvement is noted at 1-year follow-up after TB treatment initiation. **(C)** Imaging conducted 1 year after VDZ initiation shows no reactivation of the TB spondylitis. MRI, magnetic resonance imaging; TB, tuberculosis; VDZ, vedolizumab.

Three months later, an abdominal CT was performed to evaluate her CD after failed ileocolonoscopy due to poor bowel preparation. No active CD involvement was observed in the bowel, whereas a newly developed left psoas muscle abscess and spinal bony destruction were incidentally identified (Figure 3). Surprisingly, the patient did not have any back pain. Spinal MRI was conducted to confirm the diagnosis of TB spondylitis (Figure 4A). Ultrasound-guided aspiration for the paraspinal abscess was conducted, and TB PCR was positive. Abscess culture grew *M. tuberculosis* later. Bacterial and fungus culture grew no isolates. Follow-up chest CT revealed no aggravation of miliary TB. Thus, she was diagnosed with TB spondylitis.

She discontinued all medications for CD treatment and maintained TB treatment for a total of 12 months because extrapulmonary TB had developed within 3 months after the end of her miliary TB treatment. TB treatment regimen included INH, ethambutol, rifampin, and pyrazinamide for a total of 12 months. Spinal magnetic resonance imaging (MRI) at 1-year follow-up after TB treatment revealed that her spinal lesions were cured (Figure 4B).

MTX and mesalazine were restarted thereafter. However, symptoms of diarrhea and abdominal pain developed 2 months later. Her PCDAI score was 35 and the exacerbation of CD was confirmed by ileocolonoscopy. Again, acid-fast bacillus smear, culture, and PCR for TB were all negative. Due to the fear of another severe active TB with IFX treatment, vedolizumab (VDZ) was initiated instead. The patient is currently on VDZ for over a year and is maintaining clinical and biochemical remission. No serious adverse events, including TB reactivation, have occurred. Spinal MRI conducted 1 year after VDZ initiation showed no reactivation of her TB spondylitis (Figure 4C).

This case report was approved by the Institutional Review Board of Kyungpook National University Chilgok Hospital (No. 2020-09-018). All procedures performed in studies involving human participants were in accordance with the ethical standards of the institutional and national research committees and the Helsinki Declaration (as revised in 2013). Written informed consent was obtained from the patient.

## Discussion

Herein, we report a rare case of TB spondylitis in an adolescent with CD who had stopped IFX due to the development of miliary TB. This is a rare case considering that the TB spondylitis was first detected incidentally 3 months after miliary TB treatment for 12 months. This case report highlights several important points for consideration when treating patients with anti-TNF agents.

Treatment outcomes in CD have improved after anti-TNF agents have been licensed. Despite their efficacy, one of the major adverse events associated with anti-TNF agents is the risk of serious infections, including TB (9). Anti-TNF agents are associated with a 2- to 8-fold increased risk of developing active TB (9). Because most active TB cases have been reported to occur within 3–4 months after initiating anti-TNF treatment, reactivation of latent TB infections (LTBI) rather than a new infection is mainly the primary cause (9). Thus, screening for LTBI before initiating anti-TNF treatment is strongly recommended worldwide, and especially in Asia, where the prevalence of LTBI is higher than in Western countries (9). In this case report, latent TB was detected at the diagnosis of CD, and TB medication was administered for a total of 9 months.

A recent consensus from the Asian Organization for Crohn's and Colitis and Asia Pacific Association of Gastroenterology has recommended that anti-TNF treatment should be postponed for at least 3–4 weeks after commencing LTBI treatment (10). Although no large cohort studies have been investigated on the optimal time interval between the initiation of LTBI treatment and anti-TNF therapy, initiation of anti-TNF therapy in 1 month after LTBI prophylaxis in LTBI-positive patients with rheumatoid arthritis significantly reduced the risk of TB reactivation (10–12). In this case, IFX was started at 5 months after TB medication, which is a sufficient period of latent TB treatment before starting anti-TNF agents. However, miliary TB developed during treatment with IFX after 1-year treatment with IFX. Interestingly, miliary TB developed after interval shortening of IFX, which was conducted after the development of nonimmunologic pharmacokinetic loss of response. Therefore, this case highlights that miliary TB can develop during anti-TNF therapy even after a sufficient period after latent TB treatment. Moreover, caution is required when escalating the anti-TNF dose in these patients.

It is well known that when active TB is diagnosed during anti-TNF treatment, the anti-TNF agent should be withheld, and anti-TB therapy should be started (10). Regarding when to restart anti-TNF treatment, it is considered safe to delay the resumption of anti-TNF therapy until the completion of anti-TB treatment (10). However, if an early resumption of anti-TNF treatment is required, anti-TNF treatment may be restarted as early as 2 months after anti-TB treatment in patients who did not have an initially severe active TB, demonstrated a favorable response to anti-TB treatment, and when drug susceptibility was proven (10). In this case report, symptoms indicating CD exacerbation were detected in 15 months after IFX cessation and 3 months after TB treatment. Thus, we considered restarting IFX after confirmation of CD aggravation on colonoscopy. However, due to the poor bowel preparation, we failed to conduct a colonoscopy, and an abdomen CT was conducted as an alternative. TB spondylitis was detected incidentally on this scan. In this case, attempts to confirm an objective aggravation before restarting IFX eventually led to the diagnosis of TB spondylitis, and consideration of restarting IFX was withdrawn. This case suggests that an objective evaluation for unrevealed extrapulmonary TB may be required before restarting anti-TNF agents even in those who had finished a sufficient regimen of TB treatment.

TB spondylitis is a form of osteoarticular extrapulmonary TB. The disease progression is gradual and clinical symptoms such as fever, back pain, and swelling of the affected area are uncommon at the early stage of disease (5). Hence, symptoms duration until the diagnosis is long. Multiple thoracic and lumbar vertebral body involvement is common, and bony destruction can occur (5, 13). Treatment options include anti-TB medications with optional surgical interventions for spinal cord decompression and debridement. Vertebral body fusion is also considered (14). In this case, TB spondylitis in the patient was incidentally detected at an early stage when the patient did not have any relevant symptoms. Generally, the treatment duration and regimen for active TB that develops during anti-TNF therapy are the same as that for the general population (15). Thus, TB spondylitis may occur even after a sufficient duration of miliary TB treatment. Therefore, a longer period of pulmonary TB therapy may be required in patients who had previously developed pulmonary TB during anti-TNF treatment.

In adult patients with IBD, VDZ is effective for achieving clinical remission and mucosal healing (16–18). Moreover, VDZ is relatively safe compared with anti-TNF agents. VDZ did not increase the risk of serious infections, progressive multifocal leukoencephalopathy, or malignancy, according to the integrated safety data from six trials (19). TB was reported in only four patients (0.14%) with an estimated incidence of 0.1/100 PYs, among 2,830 patients with 4,811 person-years (PYs) exposure to VDZ (16). Conversely, the estimated incidence of TB during treatment with anti-TNF agents has been reported as 1.34/100 PYs and 0.79/100 PYs for IFX and adalimumab, respectively (20). Considering the complex situation of miliary TB development and detection of TB spondylitis in this case, VDZ was started instead of an anti-TNF when the patient's CD had aggravated after a sufficient treatment period for TB spondylitis. Therefore, when considering the reactivation of pulmonary or extrapulmonary TB, non-anti-TNF agents with a high safety profile may be better than anti-TNF agents, especially in complex cases like this one.

Conclusively, we report a rare case of TB spondylitis development in an adolescent with CD who had stopped IFX treatment due to the development of miliary TB and had finished a 6-month regimen of pulmonary TB treatment. Although this patient has sufficiently been treated at each stage of TB, particularly at latent and miliary TB, TB spondylitis still developed. When considering that TB spondylitis developed despite sufficient treatment at each stage, caution is required when using anti-TNF agents in patients with IBD.

## Data Availability Statement

The raw data supporting the conclusions of this article will be made available by the authors, without undue reservation.

## Ethics Statement

Written informed consent was obtained from the individual(s), and minor(s)' legal guardian/next of kin, for the publication of any potentially identifiable images or data included in this article.

## Author Contributions

JJ and SC contributed to the acquisition, analysis and interpretation of data, and drafting of the initial manuscript. YK, D-CC, SL, TP, and B-HC contributed to the acquisition, analysis and interpretation of data, and critical revision for important intellectual content. DK and BK contributed to the conception of the case report, acquisition, analysis and interpretation of data, drafting of the initial manuscript, and critical revision for important intellectual content. All authors approved the final version of the manuscript and agreed to be accountable for all aspects of the work.

## Funding

This work was supported by the National Research Foundation of Korea (NRF) grant funded by the Korean government (MSIT) (No. 2021R1A2C1011004) granted to BK.

## Conflict of Interest

The authors declare that the research was conducted in the absence of any commercial or financial relationships that could be construed as a potential conflict of interest.

## Publisher's Note

All claims expressed in this article are solely those of the authors and do not necessarily represent those of their affiliated organizations, or those of the publisher, the editors and the reviewers. Any product that may be evaluated in this article, or claim that may be made by its manufacturer, is not guaranteed or endorsed by the publisher.
